# Predictive value of inferior mesenteric artery size in type 2 endoleak after endovascular abdominal aortic aneurysm repair—a systematic review and meta-analysis

**DOI:** 10.1186/s42155-026-00715-6

**Published:** 2026-06-09

**Authors:** Galaleldin Abdelhalim, Chris Grieco, Oliver Chan, Hariesha Pathmaraj, Lexy Sorrell, Katie Carpenter, Paul Jenkins

**Affiliations:** 1 South West Imaging Training Academy, University Hospitals Plymouth NHS Trust, Plymouth, PL6 5WR UK; 2 Radiology Department, Cambridge University Hospitals NHS Foundation Trust, Cambridge, CB2 0QQ UK; 3 School of Clinical Medicine, University of Cambridge, Box 111, Cambridge Biomedical Campus, Cambridge, CB2 0SP UK; 4 Department of Interventional Radiology, Derriford Hospital, University Hospitals Plymouth NHS Trust, Plymouth, PL6 8DH UK; 5 Medical Statistics, Faculty of Health, University of Plymouth, Plymouth, PL4 8AA UK

**Keywords:** Abdominal aortic aneurysm, Type II endoleak, Inferior mesenteric artery, EVAR

## Abstract

**Background:**

Type II endoleak after endovascular aneurysm repair is the most common endoleak type. Identifying pre-operative anatomical features that could signal higher risk will improve surveillance post-procedure. This systematic review and meta-analysis evaluated the association between inferior mesenteric artery (IMA) diameter and type II endoleak.

**Methods:**

MEDLINE and EMBASE were searched via OVID (1946/1974 respectively to January 2025), in line with the PRISMA statement, for adult patients undergoing endovascular aneurysm repair for infrarenal abdominal aortic aneurysm with reported pre-operative inferior mesenteric artery diameter and post-operative type II endoleak outcomes. Both retrospective and prospective observational studies were eligible. Meta-analysis via a random-effects model evaluated the pooled mean IMA diameter among patients with type II endoleak and the mean difference in IMA diameter between patients with and without endoleak. The risk of bias was assessed using the Newcastle–Ottawa scale.

**Results:**

Twenty studies met inclusion criteria; ten provided extractable quantitative data for pooling (2176 patients; 532 type II endoleaks). Assessment with the Newcastle–Ottawa scale demonstrated that the studies had scores between 6 and 8 out of 9. The pooled mean inferior mesenteric artery diameter among cases with endoleak was 2.95 mm (95% CI 2.64–3.26 mm; *p* < 0.01; *I*^2^ = 95%). The pooled mean difference in diameter between patients with and without endoleak was 0.50 mm (95% CI 0.36–0.64 mm; *p* < 0.01; *I*^2^ = 62%), indicating larger arteries in those who developed type II endoleak. Substantial heterogeneity reflected differences in endoleak definitions, imaging protocols, and measurement methods. Subsequent sac expansion and the need for reintervention were not reported uniformly across all studies, and when reported, insufficient data were available regarding these outcomes and their relation to IMA diameter.

**Conclusion:**

Larger pre-operative inferior mesenteric artery diameter was associated with an increased likelihood of type II endoleak after endovascular aneurysm repair. However, the clinical relevance of a 0.5 mm difference remains uncertain, particularly in view of potential inter-observer measurement variability. Multicentre randomised controlled trials are needed to define actionable thresholds for treatment, considering confounding factors and clinical significance of the endoleak.

**Supplementary Information:**

The online version contains supplementary material available at 10.1186/s42155-026-00715-6.

## Introduction

Endovascular aneurysm repair (EVAR) is widely used for prophylactic treatment of abdominal aortic aneurysms (AAA) that have significant risk of rupture and mortality [1]. EVAR has a lower peri-operative mortality and complication rate in comparison to open surgical repair but is associated with higher long-term rupture and reintervention rates [2–4]. A major driver of reintervention is endoleak, defined as persistent blood flow outside the stent graft but within the aneurysm sac [5]. Endoleak can occur in 10–50% of EVAR cases, with type II endoleak (T2EL) being the most frequent subtype, observed in 8–29% of cases [5]. T2EL results from retrograde perfusion through branch vessels that are not fully excluded, such as the inferior mesenteric artery (IMA), lumbar arteries, accessory renal artery, median sacral artery, and internal iliac arteries [5].

Up to half of T2EL cases resolve spontaneously within 6 months post-EVAR, and isolated T2EL is associated with a low risk of AAA rupture (around 1%) [5, 6]. However, T2EL with sac expansion significantly increases rupture risk [7]. Prophylactic IMA embolisation may reduce the development of T2EL [8, 9], but introduces potential complications such as colonic ischaemia and the necessity of using more contrast material [10]. Moreover, increased radiation exposure to both the patient and operator is a factor to be considered, given the laterality of projection required [11]. These uncertainties call for criteria to define high-risk anatomical features, such as enlarged IMA, to guide modifications on surveillance protocols post-EVAR and potentially pre-emptive embolisation.

To address this knowledge gap, this systematic review and meta-analysis aim to quantify the association between IMA diameter and development of T2EL following EVAR. However, this must be taken in the context of other concomitant factors that may influence T2EL occurrence, such as the patency and number of other side branches, particularly lumbar arteries.

## Methods

### Search strategy

A systematic review of published work was conducted according to the Preferred Reporting Items for Systematic Review and Meta-Analyses (PRISMA) 2020 statement [12].

MEDLINE (from 1946) and EMBASE (from 1974) were searched via OVID to 01/01/2025 using the following strategy summarised in Table 1 ((‘Inferior mesenteric artery’ OR IMA) AND (endoleak? OR endo-leak? OR endo?leakage) AND (size OR diameter OR mm OR cm)).

**Table 1:** Search strategy

Abstracts or titles containing the following, limited to literature in English language						
‘Inferior mesenteric artery’			OR	IMA		
AND						
endoleak?		OR	endo-leak?	OR	endo?leakage	
AND						
size	OR	diameter	OR	mm	OR	cm

The search was limited to literature in English. Population, Exposure, Comparator and Outcome were considered as per the PECO table (Table 2). This review was not prospectively registered in PROSPERO.

**Table 2:** PECO

P	Human patients undergoing EVAR for AAA with data available on IMA diameter assessment in relation to the development of T2EL
Population	
E	Larger IMA diameter
Exposure	
C	Smaller IMA diameter
Comparison	
O	Incidence of T2EL
Outcome	

**Table:** 

*Inclusion and exclusion criteria*	
Retrospective and prospective studies on cases undergoing EVAR for AAA where the IMA diameter was assessed in relation to the development of T2EL were included. Eligible studies were required to report quantitative IMA measurements and corresponding T2EL outcomes and therefore allow comparison between patients with and without T2EL after EVAR Review articles, case reports, conference abstracts and studies that did not report the specified outcome or IMA measurements were excluded. Literature not available in English was excluded from the search	

### Study screening and data extraction

Titles and abstracts were screened independently by three authors (G.A., C.G., H.P.) to assess for suitability according to the inclusion criteria. The remaining records were then assessed for full text inclusion.

Data were extracted by five authors (G.A., C.G., H.P., P.J., O.C.). The following information was extracted from each study: first author, year of publication, country of origin, study design, follow-up time, the definition of the endoleak outcome assessed, T2EL prevalence, along with IMA diameter in those with and without a T2EL. No overlapping study populations were identified based on study centres and cohort characteristics.

### Statistical analysis

A single-group meta-analysis was conducted to estimate the pooled mean (95% CI) IMA diameter among patients with a T2EL. Subsequently, a pairwise meta-analysis was performed to assess the mean difference (95% CI) in IMA diameter between patients with and without a T2EL. A random-effects model was used for both analyses, selected based on the expectation of clinical heterogeneity between studies, with the restricted maximum likelihood estimator used to model between study variance. The impact of between-study heterogeneity was assessed visually, calculating τ^2^ and the I^2^ statistic. Analyses were conducted using the meta package [13] in R version 4.4.0 [14].

### Risk of bias

The quality of non-randomised studies was assessed using the Newcastle–Ottawa scale (Supplementary table 1) which examines patient selection methods, comparability of study groups and assessment of outcome [15].

## Results

**Fig. 1:**
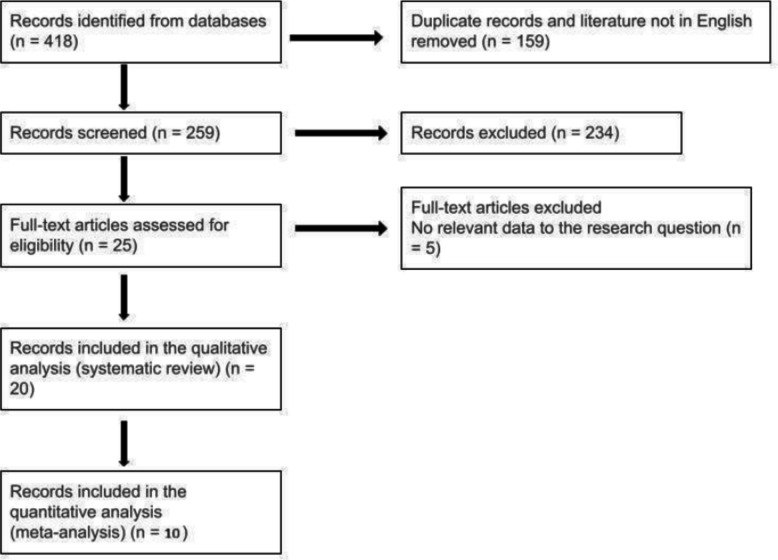
PRISMA flow diagram search results

The PRISMA flow diagram is summarised in Fig. 1. The search revealed, after exclusion of duplicates and literature not in English, 259 records to screen their abstracts and titles, out of which 234 records were excluded. Full-text articles of the remaining 25 records were retrieved and assessed. Of these, 5 were excluded due to absence of relevant data to the research question. Out of the twenty studies [16–35] included in the systematic review, however, only 10 studies had enough data to be included in the quantitative analysis [16–25]. Across the ten studies included in the meta-analysis, there were 2176 patients [16–25], of whom 532/2176 (24.4%) were T2EL-positive. Follow-up duration, number of T2EL positive and negative cases, IMA diameters, and studies’ results regarding the effect of IMA diameter on T2EL are included in Tables 3 and 4.

**Table 3:** Summary of quantitative studies’ sample sizes, IMA size and follow-up duration

**First author; year; country; study type**	**T2EL + ve n**	**T2EL − ve n**	**T2EL + ve IMA diameter (mm) mean** ± SD	**T2EL − ve IMA diameter (mm) mean ± SD**	**Follow up (months, mean unless mentioned otherwise)**
Couchet; 2015; France; retrospective [16]	84	224	3.49 ± 0.96	2.71 ± 0.73	Not reported
Liu; 2023; China; retrospective [17]	42 (T2EL-related re-intervention)	241	3.01 ± 0.46	2.63 ± 0.42	Non T2EL median 355 (IQR 162,646) days; T2EL median 178 (IQR 84,446) days
Suarez Gonzalez; 2023; Spain; retrospective [18]	16	124	3.3 ± 0.5	2.5 ± 0.5	Minimum 12
Huh; 2019; Korea; retrospective [19]	26	36	1.99 ± 1.15	1.76 ± 1.31	Median 28.00 (IQR 0.00, 167.00)
Fukuda; 2014; Japan; retrospective [20]	14 (4 persistent)	106	2.51 ± 0.21 (transient T2EL); 2.67 ± 0.06 (persistent T2EL)	2.13 ± 0.05	Not reported
Fujii; 2020; Japan; prospective [21]	131	149	2.67 ± 1.5	2.02 ± 1.6	Median 60 (IQR 24–72)
Guntner; 2014; Germany; retrospective [22]	50	116	2.8 ± 0.9	2.7 ± 0.8	Mean 28 (range 6–92)
Otsu; 2016; Japan; retrospective [23]	21 (persistent T2EL)	63	3.1 ± 0.8	2.5 ± 0.8	16.4 ± 7.7
Lowenthal; 2015; Germany; retrospective [24]	50 (persistent T2EL or requiring treatment)	80 (including 12 with transient T2EL)	3.8 ± 0.9	3.3 ± 0.7	Not reported
Chen; 2023; China; retrospective [25]	98	505	2.7 ± 1.2	2.3 ± 2.5	Median = 51 (range 5–106)

**Table 4:** Summary of quantitative studies’ IMA size effect on the presence of T2EL

First author	Effect of IMA size on T2EL	Sac expansion and/or re-intervention	Other notes
Couchet	Yes, *p* < 0.001		
Liu	Yes, *p* < 0.001	T2EL cases in this study were only T2EL related intervention cases	ROC curve identified the cutoff of the IMA diameter as 2.77 mm
Suarez Gonzalez	Yes, *p* < 0.001	Sac growth was confirmed in 10/16 of the patients with T2EL; 3/16 of them had sac increase greater than 10 mm and transarterial embolisation was performed	AUC for IMA diameter was 0.86 of endoleak development. 3.05 mm was identified as the best cutoff value; sensitivity and specificity were 77% and 86%, respectively
Huh	No, *p* = 0.463	Secondary interventions were needed in 4 patients who had expansion of the AAA sac	Differences in the size of the IMA (*p* = 0.043) between patients with (*n* = 4; 3.05 ± 0.54 mm) and without secondary intervention (*n* = 22; 1.80 ± 1.13 mm) in T2EL
Fukuda	No, *p* = 0.096	Aneurysmal sac expansion more than 5 mm during the follow up was noted in 3 out of the 4 cases with persistent T2EL and embolisation was performed via the superior mesenteric artery	
Fujii	Yes, *p* = 0.001	Older age, intraluminal thrombus volume ratio/aortic aneurysm volume and IMA diameter were statistically significant predictors of the incidence of sac expansion with persistent or new T2EL	Study also focused on sac expansion alongside T2EL
Guntner	No, odds ratio 1.14 (0.77, 1.69) (*p* = 0.51)	Aneurysm sac enlargement ≥ 5% in 16 of 24 patients with a persistent T2EL, most of them (15 of 16) had an IMA T2EL	No persistent T2EL and no aneurysm sac enlargement in patients who had preoperative IMA embolisation. No procedure-related complications during or after preoperative IMA embolisation
Otsu	Yes, *p* = 0.0083		Data extracted only relevant to persistent IMA related T2EL Circumferential thrombus protective against persistent T2EL. Number of LAs > = 1.9 mm was also risk factor for persistent T2EL along with IMA > = 2.6 mm
Lowenthal	Yes, *p* = 0.004	T2EL positive sample only includes a persistent T2EL (> 6 months) or requiring treatment	T2EL free patients included also those with transient (< 6 months and not requiring intervention) IMA average diameter different in the univariate analysis (3.3 ± 0.7 mm in the low-risk group versus 3.8 ± 0.9 mm in the high-risk group; *p* = 0.004). No statistical significance in the multivariate analysis
Chen	Yes, *p* < 0.001	T2EL was associated with the reintervention but did not affect long-term survival or increase aneurysm-related mortality after EVAR	Primary outcome was to assess the relationship between size of patent lumbar arteries with T2EL development. IMA diameter and number of lumbar arteries were independent risk factors for T2EL development

**Fig. 2:**
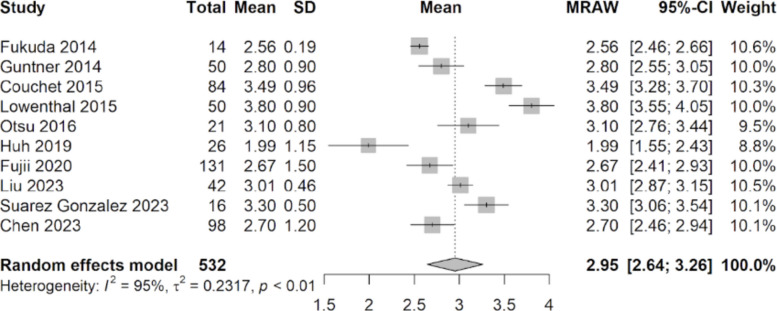
Forest plot demonstrating mean IMA diameter in cases with T2EL

The pooled mean IMA diameter among patients who developed T2EL was 2.95 mm (95% CI 2.64–3.26 mm; *p* < 0.01), with corresponding forest plot shown in Fig. 2. However, there is evidence of substantial heterogeneity between the included studies (*I*^2^ = 95%).

**Fig. 3:**
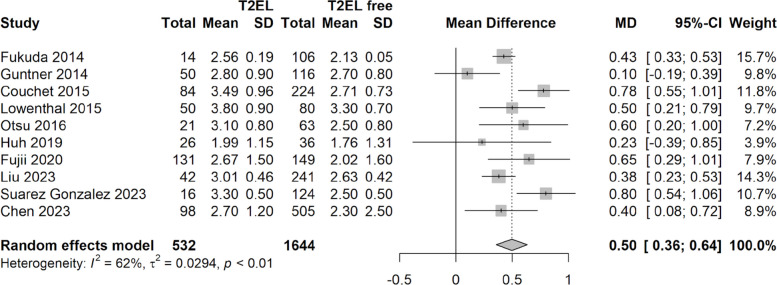
Forest plot demonstrating mean IMA diameter differences between cases with and without T2EL

The pooled mean difference in pre-EVAR IMA diameter between patients with and without a T2EL was 0.50 mm (95% CI 0.36–0.64 mm; *p* < 0.01), indicating that patients with a T2EL had, on average, a larger IMA than those who remained T2EL-free (Fig. 3). There was substantial heterogeneity (*I*^2^ = 62%).

Three out of the 10 studies included in the statistical analysis [19, 20, 22] did not find a statistically significant effect of IMA size on T2EL. However, the other 7 studies were supportive of the effect of IMA size [16–18, 21, 23–25].

In addition to the ten quantitative studies, several qualitative studies [26–34] have also been examined for the association of IMA diameter with the development of T2EL post-EVAR. They were not pooled due to heterogeneous reporting formats and incomplete numerical data, but still provide important insight into how IMA calibre, as well as other factors such as lumbar artery burden and sac thrombus volume, may influence T2EL risk. A summary is presented in Table 5.

**Table 5:** Summary of qualitative studies evaluating IMA size and T2EL

**First author; year; country; study type**	**T2EL + ve n**	**T2EL − ve n**	**Follow-up (mean unless specified otherwise)**	**Effect of IMA size on T2EL (yes/no)**	Key findings
Kondov; 2022 [26]; Germany retrospective	30 significant; 23 non-significant	224	38.9 (SD 121.6) months	Yes	Increasing IMA diameter correlated with the incidence of significant T2EL. Highest incidence of significant T2EL observed in patients with an IMA diameter measuring 3.1–4.0 and ≥ 4.1 mm (15% and 33%, respectively) Large IMA (> 3 mm) combined with > 3 patent lumbar arteries was more frequent in the group with a significant T2EL than in the other patients: 80.0% versus 4.9%, *p* < 0.0001
Aoki; 2017 [27]; Japan; retrospective and prospective	24	18	0.23 months (1 week)	Yes	T2EL was more common with an IMA diameter ≥ 2.5-mm diameter than in those with IMA < 2.5-mm 3/16 (18.8%) vs. 21/26 (80.8%), respectively; *p* < 0.0001
Sampaio; 2005 [28]; USA; retrospective	41	137	Median 12 months (range 1–65)	No	No relevance of IMA diameter to T2EL Ostial thrombus was protective (OR 2.3; *p* = 0.0001)
Walker; 1998 [29]; UK; prospective	5	42	Not reported	No	5 patients developed T2EL (all lumbar origin). Among 42 without T2EL, 5 had patent IMAs pre-EVAR yet no leaks
Zhou; 2014; USA; retrospective	34	149	53 months	No	34/183 (18.6%) developed T2EL. No significant correlation between the diameter of IMA or lumbar arteries and sac enlargement among T2EL cases
Spanos; 2020 [31], Greece; retrospective	82 persistent T2EL	58 temporary T2EL	12 months	No	Study compared 2 groups which had endoleaks, of which 82 had persistent T2EL, and the remaining 58 T2EL patients resolved without reintervention. There was no difference in IMA diameter between groups
Arko; 2003 [32]; USA; retrospective	16 persistent T2EL	14 temporary T2EL	29.9 ± 7.9–30.2 ± 8.6 months	No	Small single-centre study that found no significant relationship between IMA size and the persistence of T2EL at 6 months
Müller-Wille; 2015 [33]; Germany; retrospective	23 Sac enlargement	33 No sac enlargement	36 ± 24 months	Yes	Larger IMA diameter in patients with aneurysm enlargement compared to those without enlargement (mean, 3.5 mm ± 0.9 [range, 2.2–5.0 mm] vs 2.7 mm ± 0.6 [range, 1.7–3.8 mm], respectively; OR = 4.24; *p* = 0.02)
Piazza; 2017 [34]; Italy; retrospective	66	123	38 months (1–96)	No	IMA > 3 mm was not demonstrated as a statistically significant independent risk factor for T2EL development. However, cases with multiple aortic side branches with or without an IMA > 3 mm were found to be associated with more T2EL and T2EL related reintervention
Samura; 2018 [35]; Japan; retrospective	48	148	21.0 ± 16.9	Yes	Receiver operating characteristic (ROC) curve analysis, indicated that patent IMA diameter ≥ 3.0 mm (OR, 4.09; 95% CI, 1.04–9.65; *p* = 0.043), was a significant risk factor for T2EL. For 3 mm, sensitivity and specificity were 47.6% and 85.4%, respectively)

Few studies from data included in the qualitative analysis demonstrated correlation between a larger IMA diameter (≥ 2.5–3.0 mm) and aneurysm sac enlargement [26, 27, 33]. Large IMA (> 3 mm) together with more than three patent lumbar arteries or multiple abdominal aortic side branches was more frequent in the T2EL group [26, 34]. Ostial thrombus was reported to be a protective factor against T2EL development [28].

Conversely, several cohorts did not find a statistically significant association between IMA size and T2EL development or persistence [28–32, 34].

## Discussion

Our meta-analysis shows that patients who developed T2EL had a mean pre-EVAR IMA diameter of 2.95 mm, and that IMA diameter was on average 0.50 mm larger in patients with T2EL than in those without. Although significant heterogeneity limits our ability to define a specific threshold above which T2EL risk markedly increases, these findings shed some light on the potential influence of IMA size on T2EL post-EVAR. However, the pooled mean IMA diameter does not account for baseline differences in study demographics nor other potential confounding factors.

Although this review focused on IMA diameter, several included quantitative cohorts also reported that the lumbar artery burden, such as the number of patent arteries or their diameter, was associated with T2EL, with an effect of similar significance to that of IMA diameter. Lumbar arteries may sustain retrograde inflow into the aneurysm sac, particularly when multiple vessels are present. These observations support interpreting IMA diameter as one metric of a broader anatomical risk profile, rather than a stand-alone endoleak determinant.

Several meta-analyses report that prophylactic aortic side branch embolisation including IMA embolisation is associated with fewer T2EL and fewer reinterventions [10, 36, 37]. However, the evidence remains heterogeneous, lacking a definitive consensus on who benefits the most from prophylactic IMA embolisation. This review offers a pragmatic approach: if prophylactic embolisation is considered, it should be reserved for patients with high-risk anatomical characteristics, rather than as a universal adjunct to EVAR. Further trials to evaluate the risk profile of IMA embolisation and to quantify the benefit are required [38].

Within the literature concurrent IMA embolisation is generally feasible with a high success rate, but adds complexity that increases procedure time, contrast use, radiation exposure, and device-related costs [37]. The main concern is bowel ischaemia, particularly in patients with compromised collateral perfusion. Nevertheless, the available evidence suggests that even though bowel ischaemia is a potentially devastating complication, interruption of the IMA appears not to be a major driver of this extremely rare post-EVAR complication [39, 40].

Cost implications are also uncertain. One retrospective cohort study found that the upfront procedural costs in preventative IMA embolisation managed to offset the potential downstream costs of managing clinically significant endoleak, imaging burden, and reintervention [41]. Regardless, robust health-economic evaluations are limited, and future work needs to assess whether selective prophylactic embolisation is cost-effective over a lifelong EVAR surveillance pathway.

Current guidelines support lifelong imaging surveillance after EVAR, with increasing use of duplex ultrasound when the first-year assessment shows no endoleak or sac expansion [2, 42]. While the presence of endoleak is a main driver for the decision to pursue closer follow-up, larger IMA diameters may be considered as additional surveillance stratifiers. Patients with larger pre-operative IMA diameters, particularly when combined with other anatomical risk factors, may warrant greater attention on follow-up imaging. This ‘precision surveillance’ approach fits guideline principles while still recognising that many T2ELs remain benign and surveillance burden should be proportional to the likelihood of significant progression [5, 43].

Several limitations should be considered. Firstly, most included studies were retrospective single-centre cohorts. Non-standardised imaging protocols pose a potential challenge. For instance, Otsu et al. followed up cases with multiphase computed tomography angiography (arterial and delayed phases) [23], this raises the possibility of endoleaks being overlooked in cases without a multiphase imaging protocol. There is also heterogeneity in how T2EL was defined; Liu et al. only included reintervention-requiring cases [17].

Löwenthal et al. compared a low-risk group (without T2EL or with transient T2EL) to a high-risk group (persistent T2EL or need for reintervention) rather than the absolute presence or absence of T2EL [24]. Otsu et al. assessed T2ELs which persisted for at least 6 months post-EVAR [23]. IMA measurement protocols, ostial stenosis and thrombus, presence of different causes of endoleak and duration of follow-up may also be of significant influence. Our study also overlooks the impact of other potentially confounding factors such as other patent large side branches, anticoagulants and sac expansion.

Importantly, our findings support a selective rather than universal preventative strategy, encouraging clinicians to consider IMA diameter as one component of T2EL risk stratification, alongside other anatomical and patient-specific factors. Future high-quality randomised controlled multicentre studies are needed to confirm whether a particular diameter of IMA is associated with high risk of T2EL. Establishing this relationship will enable more personalised procedural planning, reducing endoleak complications and the need for reinterventions, ultimately improving long-term EVAR outcomes and patient safety.

## Conclusion

IMA diameter was consistently larger in patients who developed T2EL, with an average difference of 0.5 mm. Significant heterogeneity limits our interpretation of a 2.95 mm mean IMA diameter in T2EL cases. Further multicentre randomised-controlled trials would be crucial to define anatomical thresholds that reliably identify clinically significant T2EL, taking into consideration other potential confounding factors such as the patency and calibre of other aortic side branches.

## Supplementary Information

Supplementary Material 1: Table 3.

## Data Availability

All data generated or analysed during this study are included in this published article and its supplementary information files.

## Abbreviations

EVAR: Endovascular aneurysm repair
AAA: Abdominal aortic aneurysm
T2EL: Type II endoleak
IMA: Inferior mesenteric artery
